# Supplementary figures and images for: Estimating neuronal firing density: A quantitative analysis of firing rate map algorithms
Source: PLoS Comput Biol. 2023 Dec 27;19(12):e1011763. doi: 10.1371/journal.pcbi.1011763 (PMC10775984; doi:10.1371/journal.pcbi.1011763)

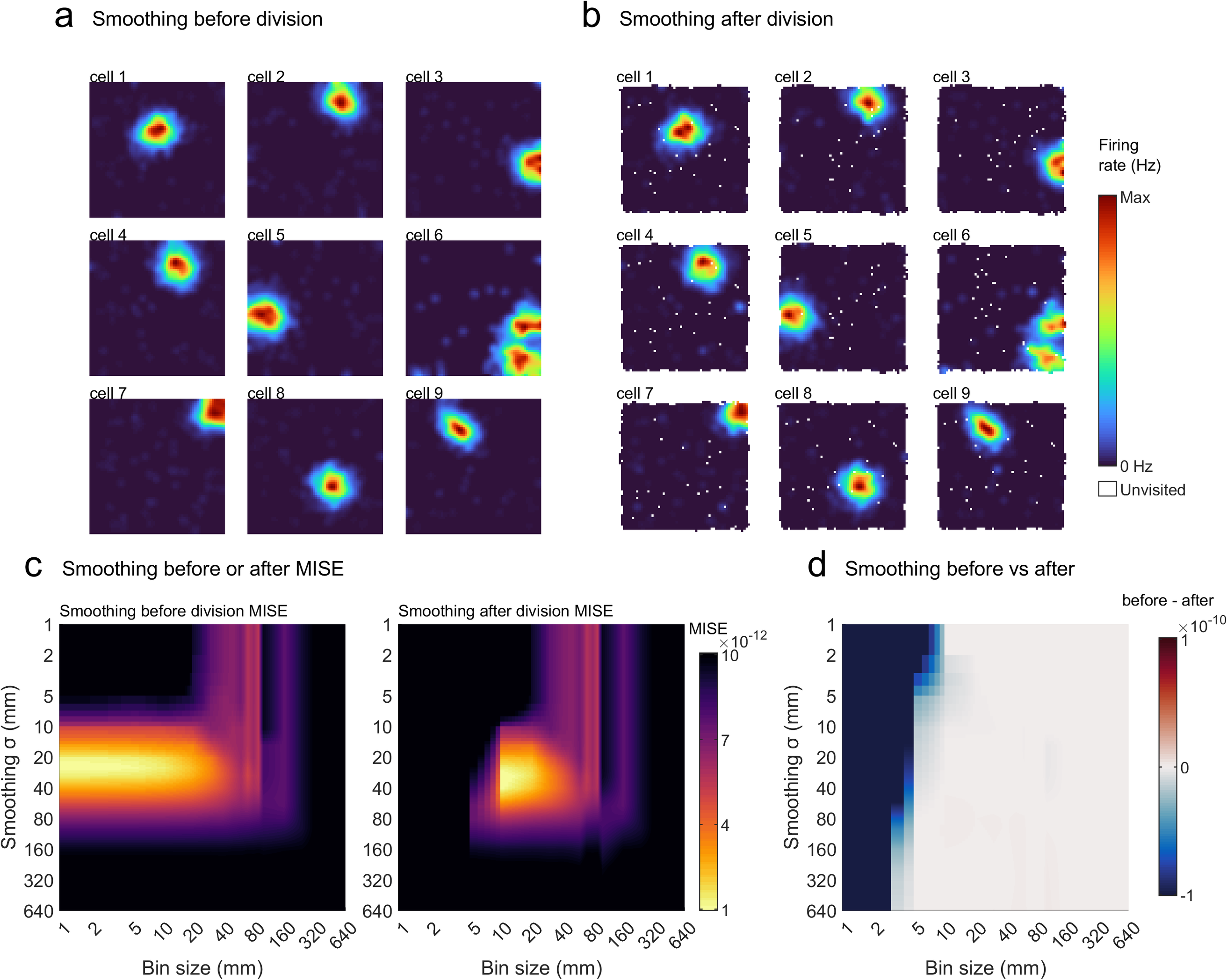

Supplement: S1 Fig — A) Example firing rate maps where smoothing was applied to the spike and dwell maps before division. B) Same as a but these maps were created by smoothing the rate map after division. Note the greater number of empty bins. C) MISE maps for the approaches in a and b respectively, shown as in Fig 7. d) Smoothing before division error map minus the smoothing after error map. Smoothing before division is less accurate at small bin sizes, the two approaches are comparable otherwise. (TIF) [file pcbi.1011763.s001.tif]

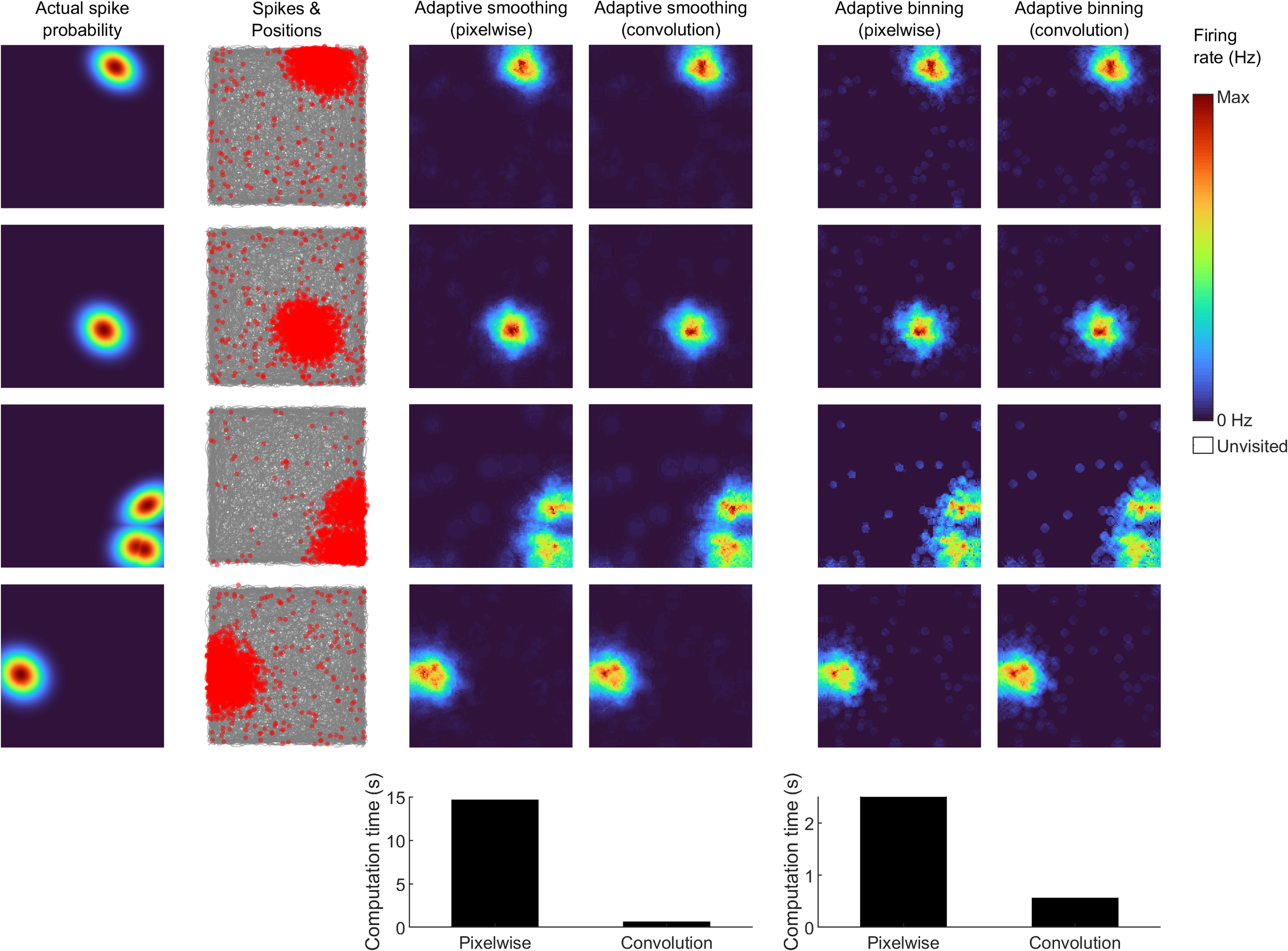

Supplement: S2 Fig — Four example cells, one per row. Columns show each cell’s spike probability map, simulated spikes and trajectory, firing rate map generated using the pixelwise adaptive smoothing approach described by Skaggs and McNaughton [42], a convolution implementation of this, the pixelwise adaptive binning approach described by Yartsev and Ulanovsky [44], and a convolution implementation of this. Bar graphs show the average time taken to generate the maps in each column. (TIF) [file pcbi.1011763.s002.tif]

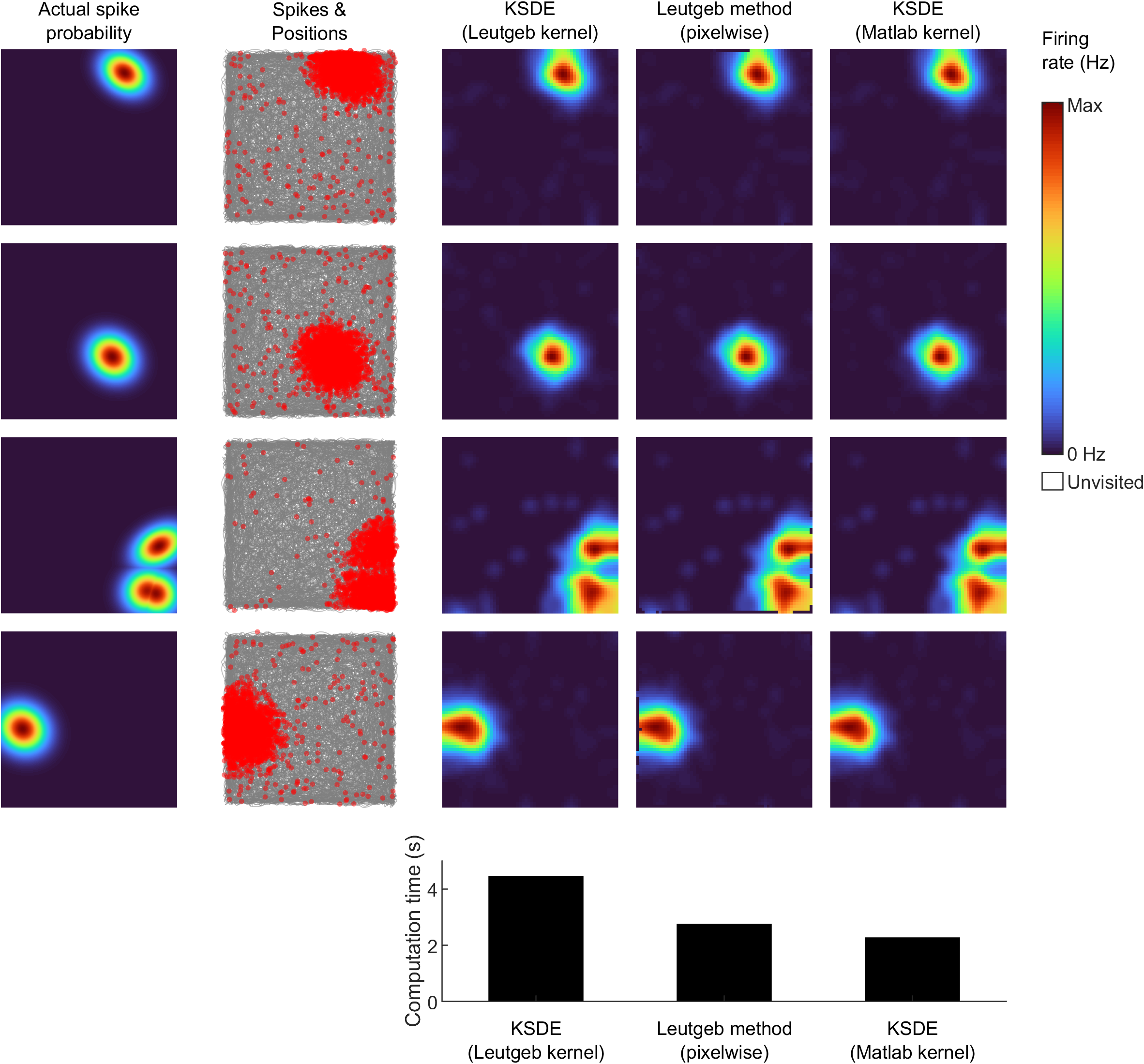

Supplement: S3 Fig — Four example cells, one per row. Columns show each cell’s spike probability map, simulated spikes and trajectory, and three firing rate maps generated using a MATLAB implementation of the kernel described by Leutgeb et al. [50,51], a pixelwise implementation of this (i.e. as described in the literature) and an implementation using the built-in MATLAB mvksdensity function and its kernel respectively. Bar graphs show the average time taken to generate the maps in each column. Because the last implementation was the fastest and did not visibly differ from the others, we used that approach. (TIF) [file pcbi.1011763.s003.tif]

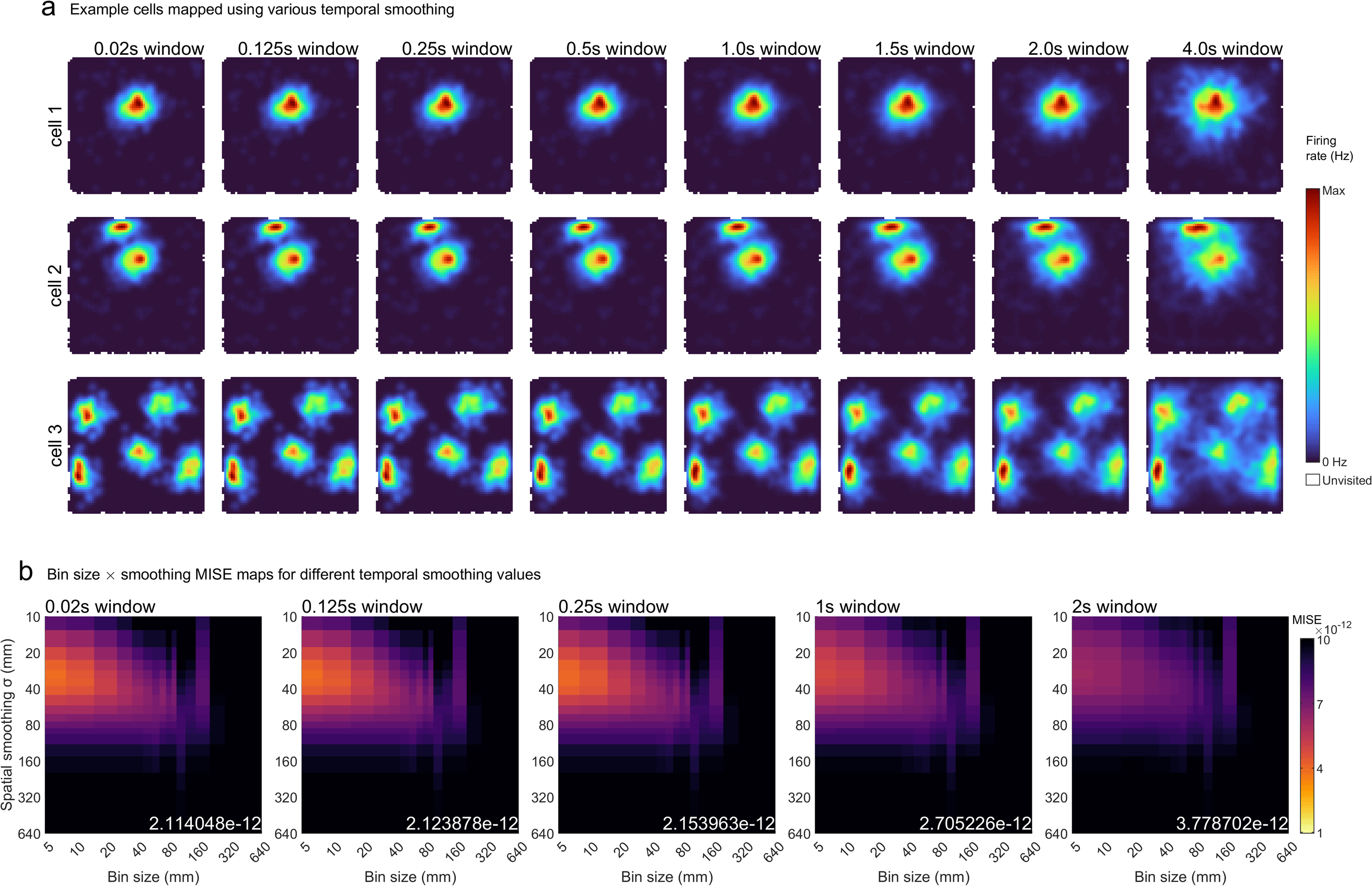

Supplement: S4 Fig — A) Three example cells, one per row. Columns show firing rate maps generated using the temporal KSDE approach (bin size = 20 mm, spatial smoothing = 30 mm) with increasing temporal smoothing window durations. B) MISE maps for increasing temporal smoothing window durations. Bottom right text gives the minimum MISE value in each map. The color axis used here does not show the full range of data values but was chosen to visually isolate any low error region(s). (TIF) [file pcbi.1011763.s004.tif]

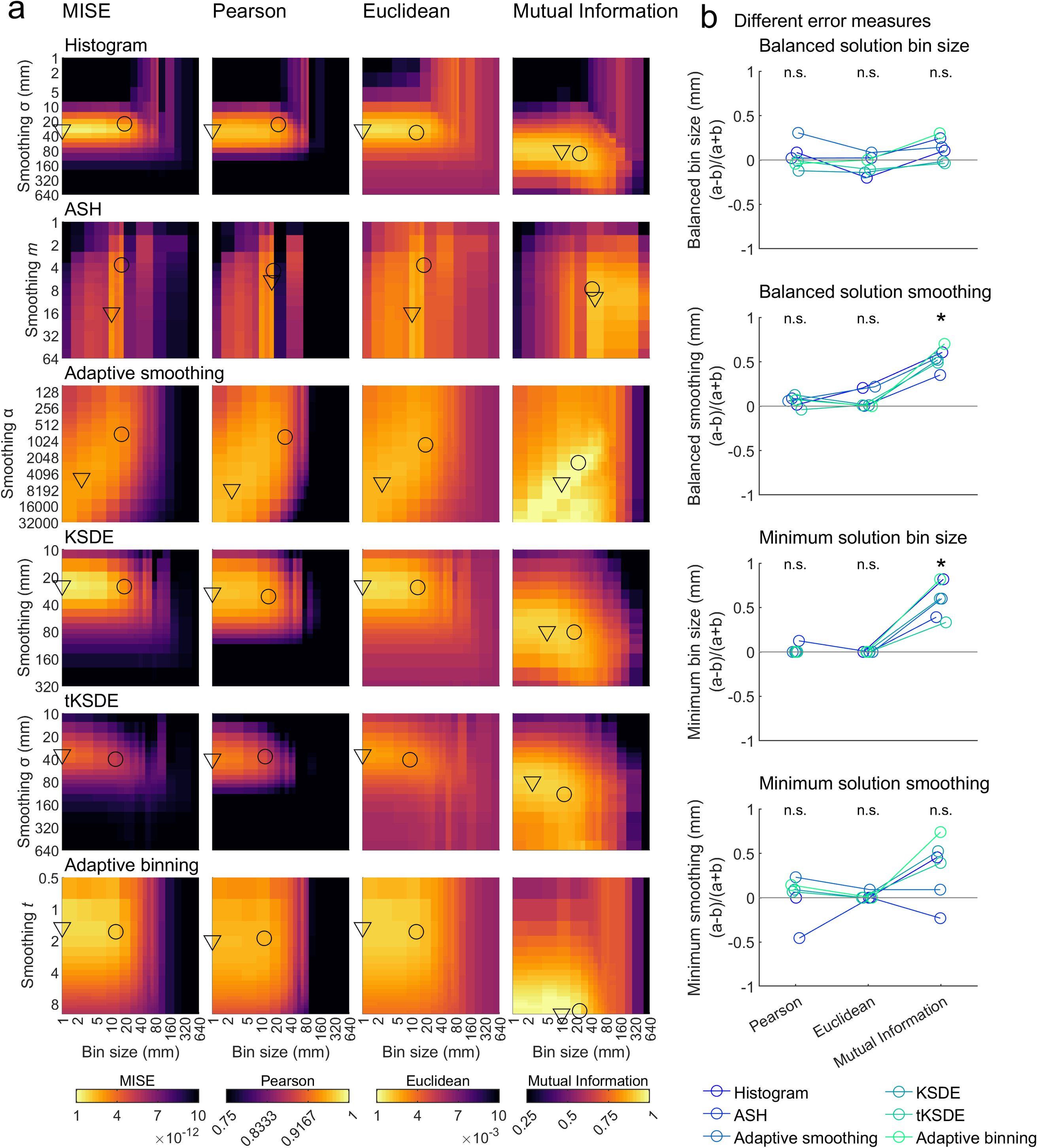

Supplement: S5 Fig — A) Error plots for 64 cells simulated in 8-minute sessions for each mapping method (rows) and for multiple error measures (columns), from left to right: mean integrated squared error (MISE), pairwise Pearson correlation, Euclidean distance and Mutual Information (Matlab function mi, J. Delpiano). There is little difference between the measures. Note that plots are shown using a consistent color axis which may not span the full range of data values for every method. B) Top: the balanced mapping solution bin size for each mapping approach and error measure, normalized relative to MISE. Text above gives the result of a one-sample t-test comparing each group to zero (* = p < .05, n.s. = not significant). Plots below show the same but for the balanced solution smoothing strength, minimum solution bin size and minimum solution smoothing strength. For visualization, a small amount of jitter was added to the x-axis values in these plots. (TIF) [file pcbi.1011763.s005.tif]

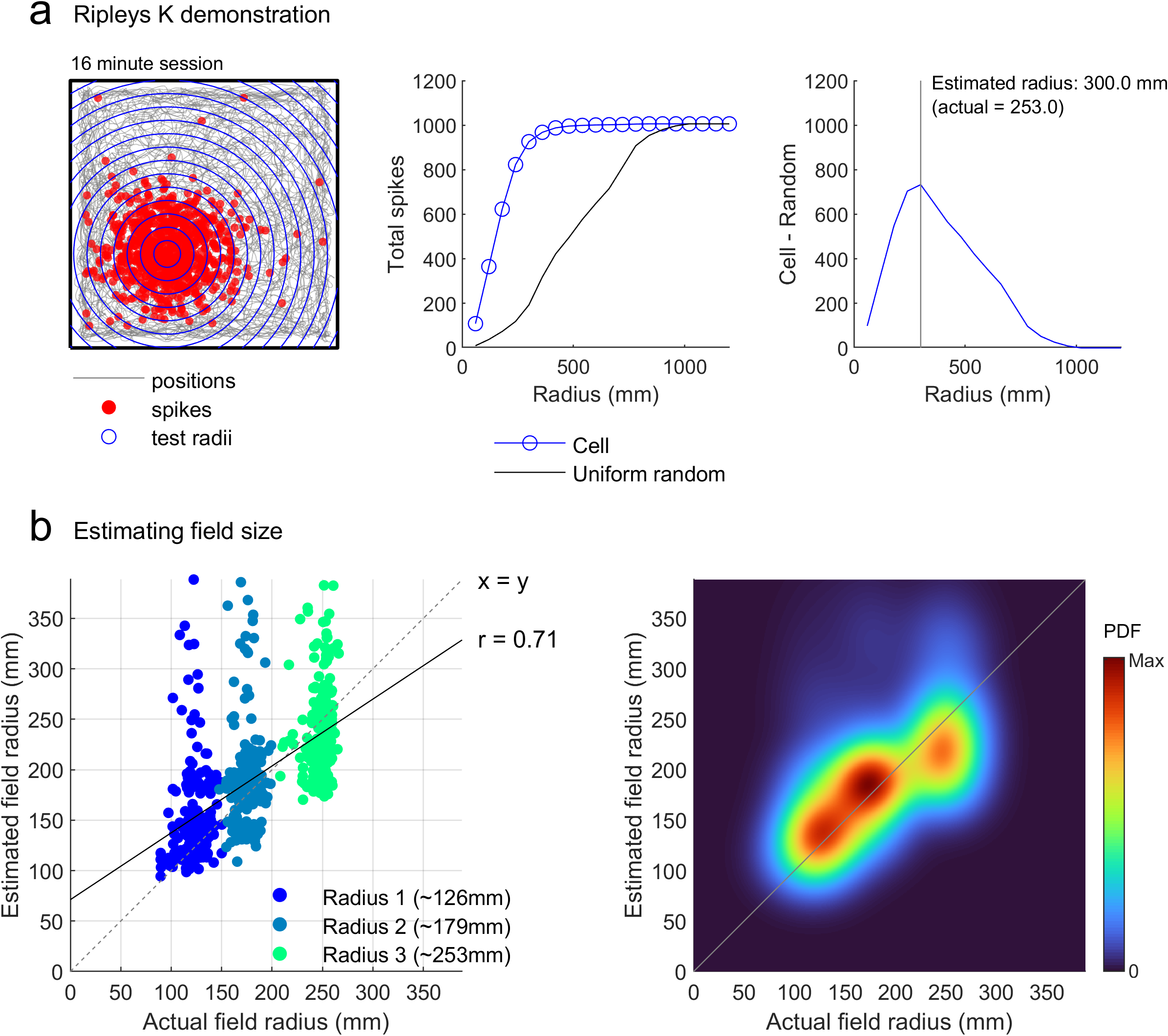

Supplement: S6 Fig — A) Left: The spikes and trajectory of an example simulated place cell. Overlaid are test radii of increasing size. Middle: for each radius, the total number of spikes that fall within its area (blue line) and the number of spikes we would expect if they were distributed uniformly across the animal’s trajectory (black line). Right: the number of spikes minus the expected number and the maximum of this function. The maximum is related to the spatial clustering or place field size of the place cell. Ripley’s K function is related to this example, but it is repeated across many different locations in the environment and the results are averaged. B) Left: the result of applying the Ripley’s K procedure to all our simulated place cells, colors denote the three firing field size groups (Fig 1). Black line shows a linear fit to the data (Spearman’s correlation between estimated and real field size: r = 0.71, p = 3.6 × 10−118). Gaussian jitter (mean = 0, σ = 8) was added to the y-values to make visualization of overlapping data clearer. Right: kernel smoothed probability density estimate of the data (no jitter; Matlab ksdensity with 25 mm bandwidth). (TIF) [file pcbi.1011763.s006.tif]

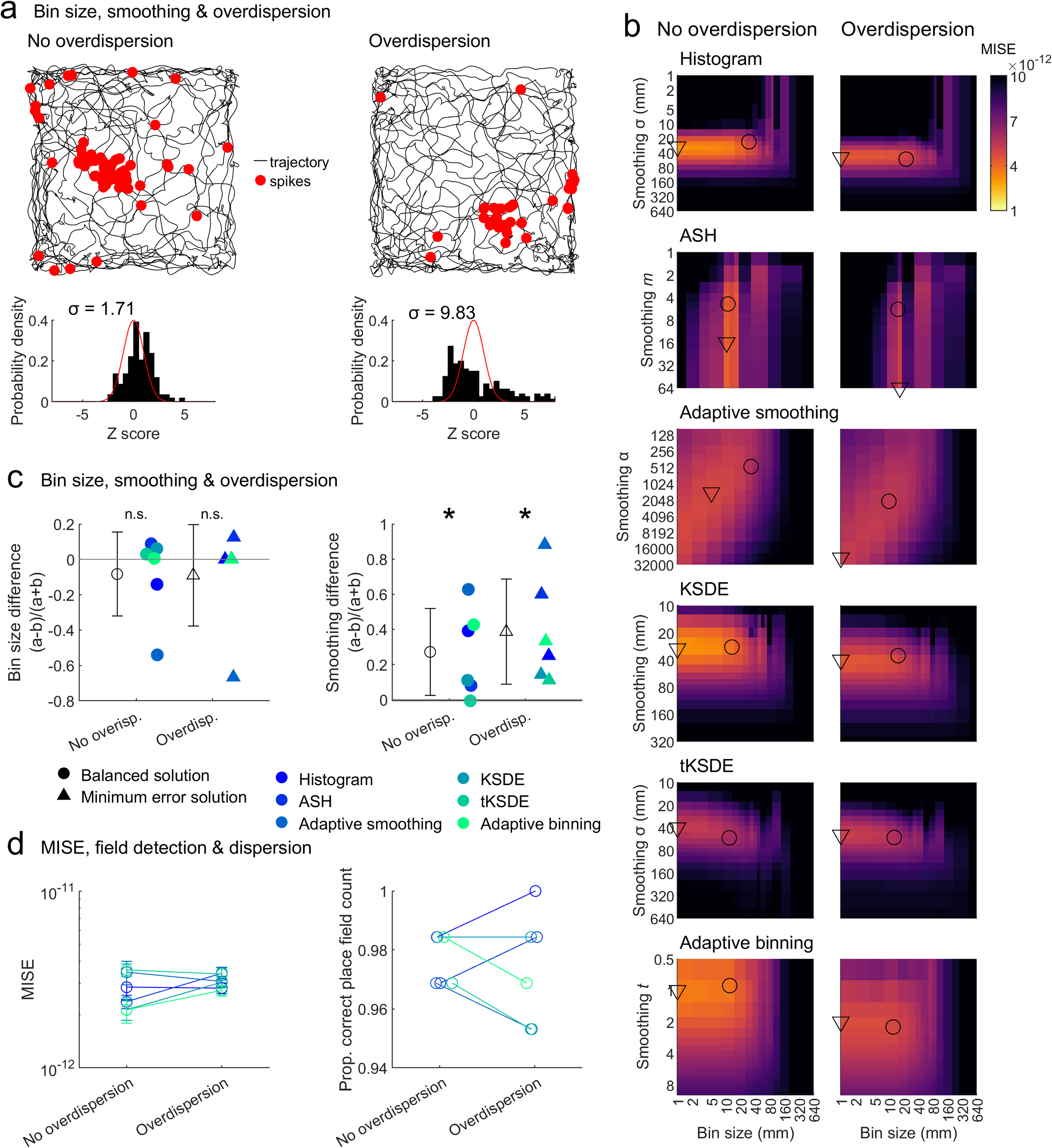

Supplement: S7 Fig — A) Two example cells, one per column. The left cell’s spiking activity does not include overdispersion, the bottom plot shows the variability in the cell’s firing during passes through the place field (black bars; text above gives the standard deviation of this distribution) which matches closely the expected distribution if the cell was modulated by space alone (red line). The right cell’s spiking does include overdispersion. B) The MISE error plots for 64 cells simulated in 4-minute sessions without overdispersion (left column) and with overdispersion (right column) for each mapping method (rows). There is very little difference between the columns. Note that plots are shown using a consistent color axis which may not span the full range of data values for every method. C) Left: balanced and minimum error solution bin sizes did not change when overdispersion was included. Right: balanced and minimum error solution smoothing strength tended to be significantly higher when overdispersion was present. Text above gives the result of a one-sample t-test comparing each group to zero (* = p < .05, n.s. = not significant). D) The MISE (left plot) and error in detecting place fields (right) did not differ when overdispersion was present if using the balanced solution. For visualization, a small amount of jitter was added to the place field plot’s x-axis values. (TIF) [file pcbi.1011763.s007.tif]

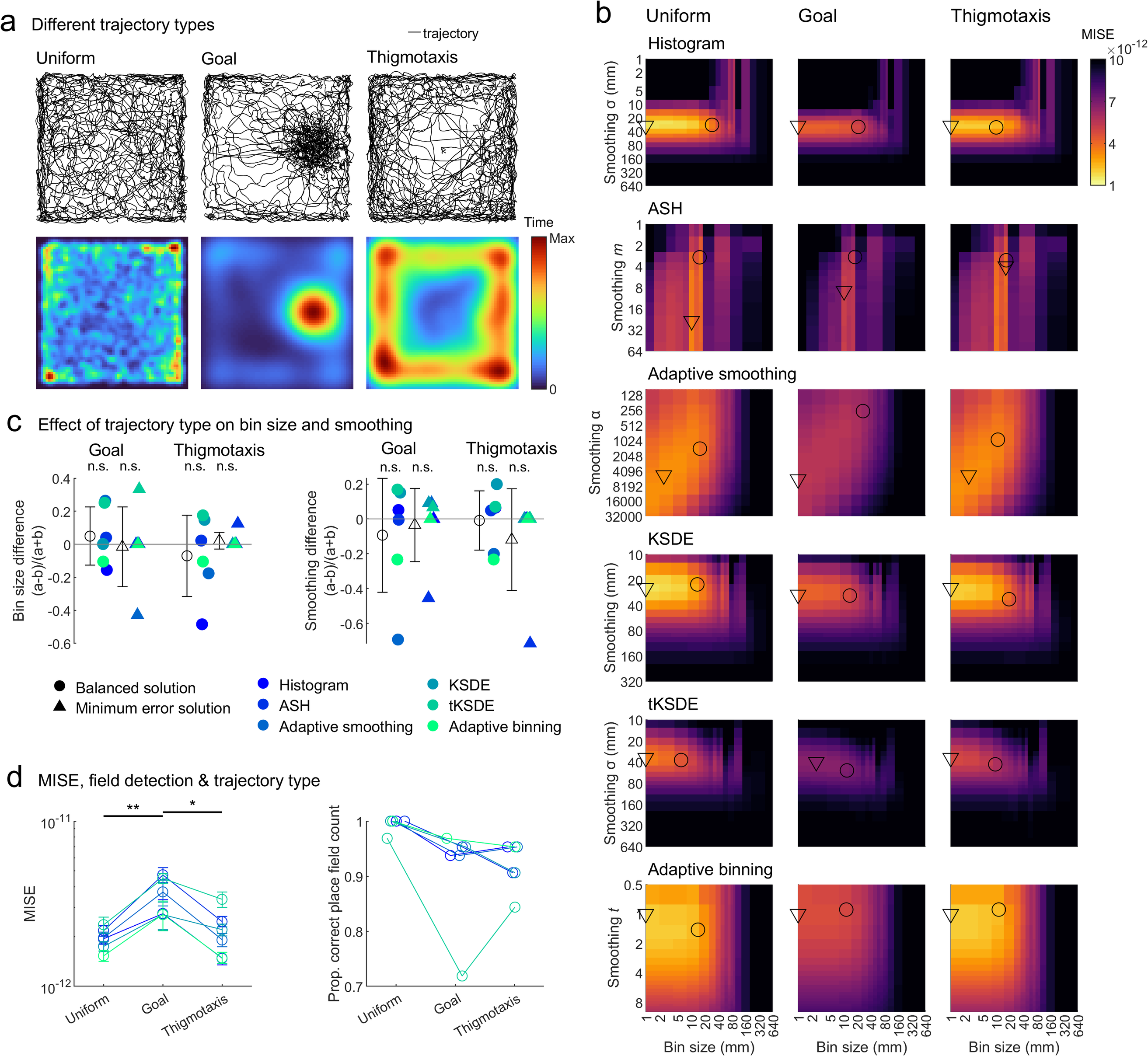

Supplement: S8 Fig — A) Three example trajectories, 8-minutes long, one per column, from left to right: uniform sampling of the environment, a strong bias to one location such as a goal and a bias for moving close to the walls. Plots below show the dwell time map for each trajectory. B) The MISE error plots for 64 cells simulated in 8-minute sessions, for each of the trajectory types (columns) and for each mapping method (rows). There is very little difference between the columns. Note that plots are shown using a consistent color axis which may not span the full range of data values for every method. C) Left: balanced and minimum error solution bin sizes did not change when trajectories were biased. Values are normalized relative to the ‘uniform’ trajectory results. Text above gives the result of a one-sample t-test comparing each group to zero (* = p < .05, n.s. = not significant). Right: same for smoothing strength. D) The MISE (left plot) and error in detecting place fields (right) when using the balanced solution for each trajectory type. MISE increased for the ‘goal’ trajectory, which can also be seen in the middle column of panel b, which is darker than the others. For visualization, a small amount of jitter was added to the place field plot’s x-axis values. (TIF) [file pcbi.1011763.s008.tif]

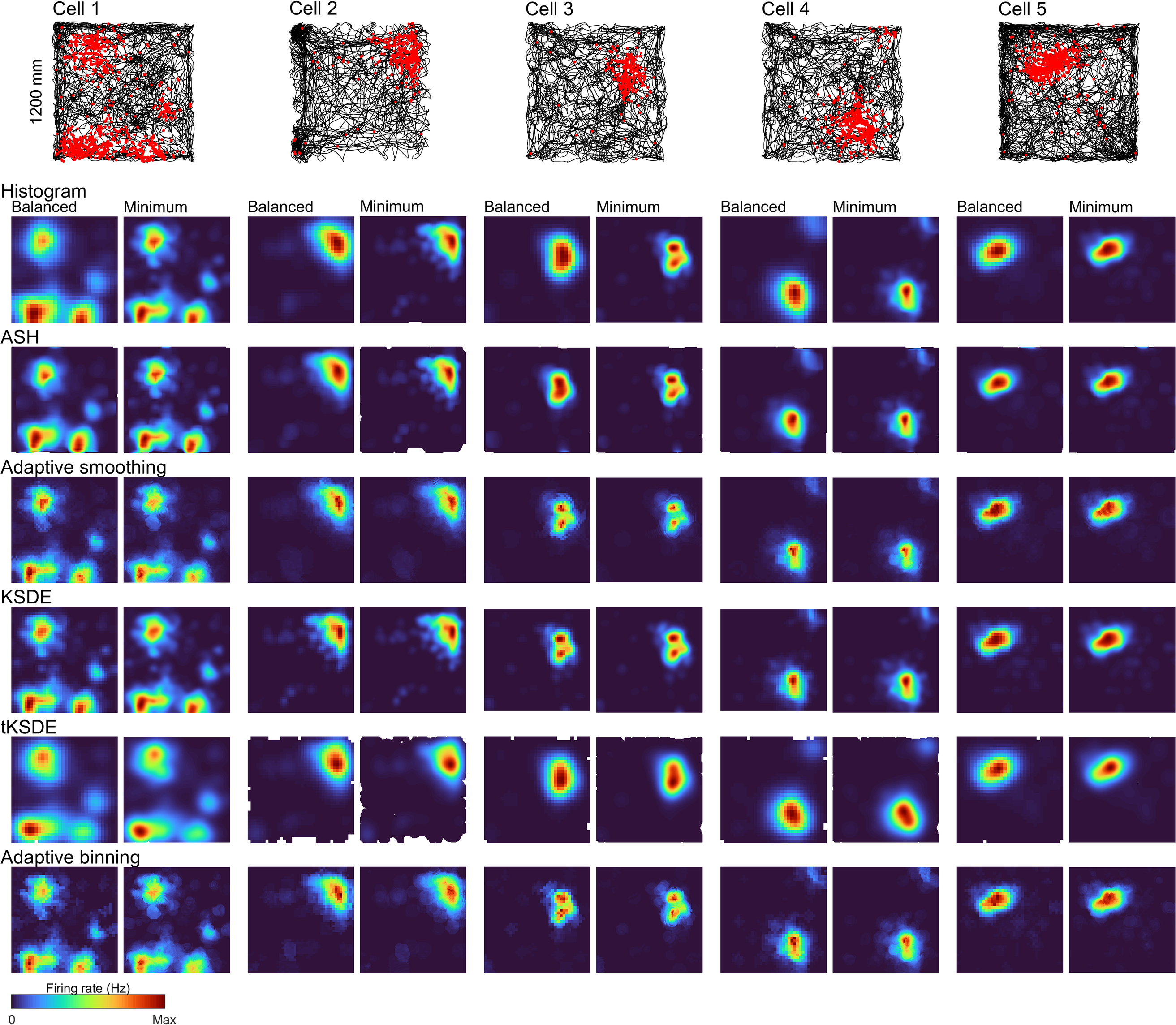

Supplement: S9 Fig — Top row shows the spike (red dots) and position data (black line) for 5 place cells from the dataset of [20] available online. Below each of these, firing rate maps are shown that were generated using the balanced solution parameter equations shown in Table 1 (left column of each cell) and maps generated using the minimum error solution parameter equations shown in Table 1 (right column of each cell). In both cases parameters were calculated assuming an average field radius of 300 mm and a recording duration of 25 mins. One mapping method is shown per row. (TIF) [file pcbi.1011763.s009.tif]

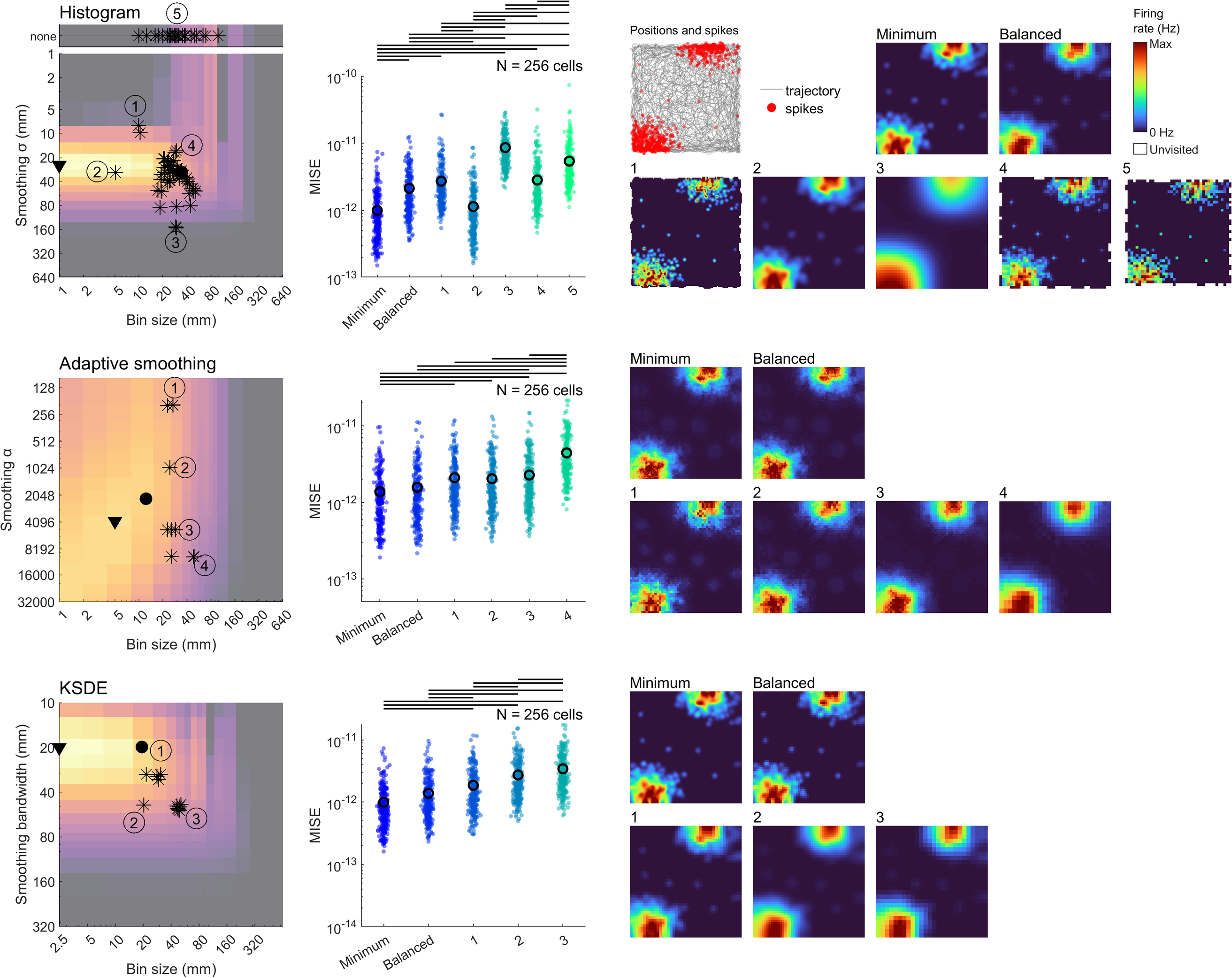

Supplement: S10 Fig — Related to Fig 11B. Rows show the results for the Histogram, Adaptive smoothing and KSDE mapping methods respectively. Left column: MISE map overlaid with the minimum error, balanced and literature reported parameter combinations as in Fig 11B. Gaussian jitter (mean = 0, σ = 2) was added to the literature values to make visualization of overlapping data clearer. For each mapping method 3–5 literature values have been selected and numbered at points of interest. Middle column: the MISE associated with the minimum error and balanced parameter combinations and the numbered points in the MISE map for all 256 simulated place cells. In each plot groups differed significantly (Histogram: F(6,1785) = 167.9, p = 1.93×10−169; Adaptive: F(5,1530) = 73.6, p = 3.36×10^−69^; KSDE: F(4,1275) = 74.5, p = 8.06×10^−57^; one-way ANOVA) and horizontal lines denote a significant (p < .05) post-hoc comparison. MISE values vary greatly across the different literature values and the numbered points are generally less accurate than the balanced solution. Right column: firing rate maps generated for an example place cell using the minimum error and balanced parameter combinations and the numbered points in the MISE map. While some parameter combinations produce maps similar to the minimum error or balanced solution, they often deviate significantly. (TIF) [file pcbi.1011763.s010.tif]

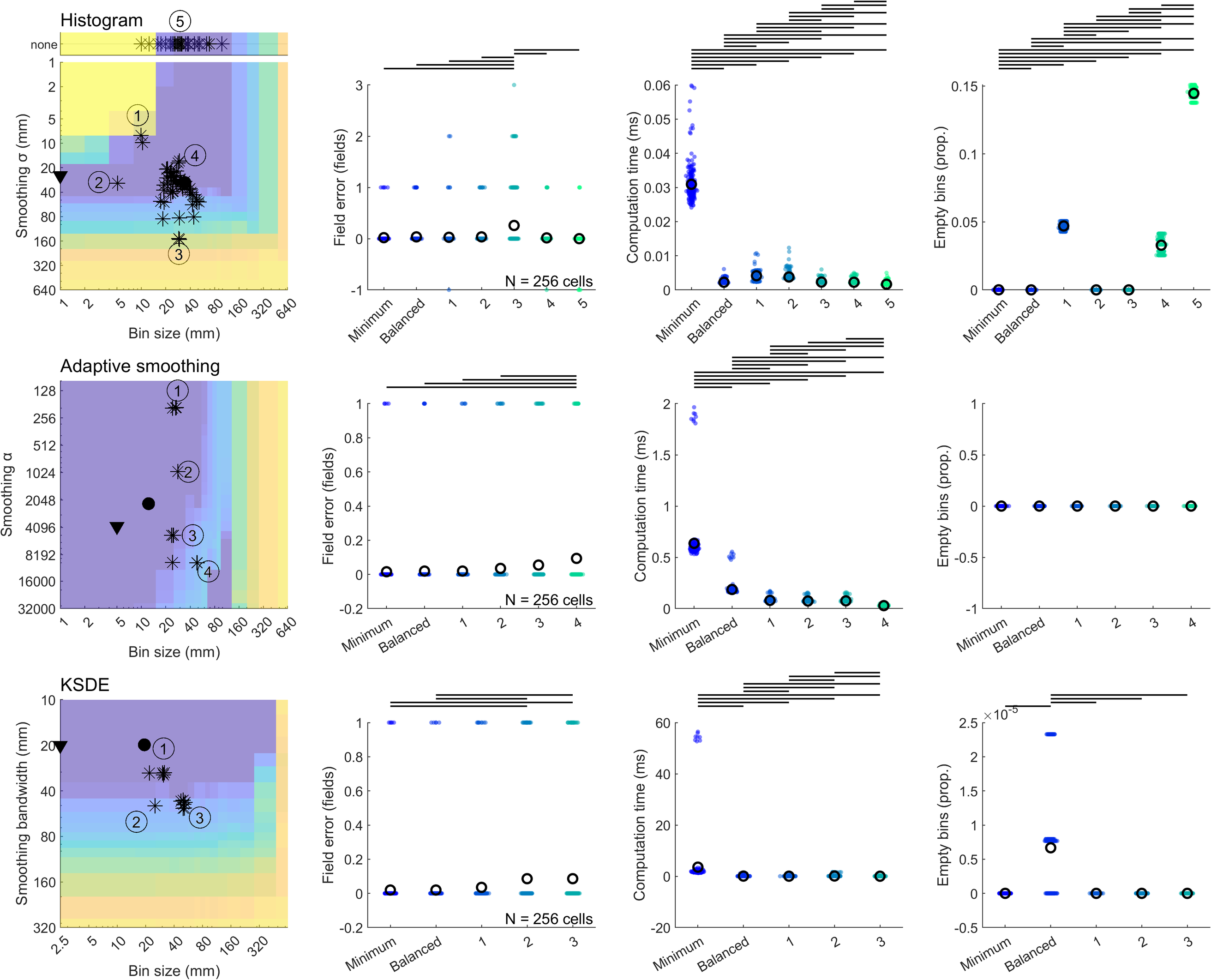

Supplement: S11 Fig — Related to Figs 11B and S10. Rows show the results for the Histogram, Adaptive smoothing and KSDE mapping methods respectively. Left column: place field detection error map overlaid with the minimum error, balanced and literature reported parameter combinations as in Fig 11B. Gaussian jitter (mean = 0, σ = 2) was added to the literature values to make visualization of overlapping data clearer. For each mapping method 3–5 literature values have been selected and numbered at points of interest. Right columns: the field detection error, computation time and proportion of empty bins respectively associated with the minimum error and balanced parameter combinations and the numbered points for all 256 simulated place cells. Horizontal lines denote a significant (p < .05) post-hoc comparison following a significant Kruskal-Wallis omnibus test. (TIF) [file pcbi.1011763.s011.tif]
