# Supplementary material for: Estimating neuronal firing density: A quantitative analysis of firing rate map algorithms
Source: PLoS Comput Biol. 2023 Dec 27;19(12):e1011763. doi: 10.1371/journal.pcbi.1011763 (PMC10775984; doi:10.1371/journal.pcbi.1011763)
Supplement: Attachment — Submitted filename: reviewPcb.pdf [file pcbi.1011763.s013.pdf]

This manuscript reviews existing and new methods and parameters used to generate firing rate maps of spatially modulated neural activity. In particular, the author thoroughly explores the impact of those methods and choice of parameters on how the rate maps correspond to the underlying spike distribution, which is derived from simulated data.

Overall, the analysis is rigorously performed, and the results convincingly demonstrated. The comparisons performed in the paper are informative for the spatial cognition field. However, I have concerns about the novelty and the impact of the results of this paper. In terms of novelty, the author showed that the bivariate histogram with spatial smoothing was the preferred choice in the majority of cases, but this is already the most commonly used method to compute rate maps. Similarly, in terms of impact, the author shows a method to optimise the parameters used when computing rate maps, but those optimal parameters are in the range most commonly used in the literature. Furthermore, the fact that this study is only performed on synthetic data makes it difficult to gauge the efficiency of this method on real datasets.

Major:

- The way the author quantifies the rate map error is difficult to reconcile with a tangible measure. It would be helpful to provide other measures of similarity (correlation, mutual info, population vector distance, …) between the 'true' rate map and the computed rate maps. In its present form, the MISE is adequate to investigate the point that the author is making (eg: best method and parameters for rate map computation) but it is difficult to relate the MISE and thus the variation of method and parameters to concrete and more classic similarity measures. The author uses the place field detection, compute time, and proportion of empty bins, but adding other measures, classically used in the literature would strengthen the message and scope of the method detailed in this paper. In its present form, I am circumspect about the impact of these optimisations on a real dataset.
- This analysis is performed on synthetic data, where the author fitted as much as possible various parameters of real datasets (see methods). However I am concerned about the robustness of these results on real datasets. Notably, with different place field size (more variation than presented in this paper), different geometry of the environment (linear/star mazes, multiple rooms, very large environments....). The demonstration of the best method to bin spike data needs a synthetic dataset in order to access the underlying spike probability. However, to optimise for the detection of place fields, compute time, and empty bins (fig 10-13), in some cases, the author does not necessarily need the spike probability distribution. In these cases, it would be interesting and easy for the reader to grasp the effect of the parameter optimization on a real word dataset. In a real dataset, the place field size, number, firing rate and distribution will

vary much more than for the simulated place cell population of this paper (random distribution of place fields , same firing rate, different place field size in a single cell,...). All these discrepancies, notably the difference in firing rate and size of place fields have the potential to strongly influence the optimal parameter choice (optimal smoothing,...).

- 46 “These parameters can have a huge impact on the map created”, The author shows that this is the case in theory. However, the author shows in Fig 14 that for previous work the solution is very close to the balanced solution. I thus wonder how big is the difference in place field detection, MISE,... in the range of parameters classically used in the field. According to figure 13, the difference might be small.

<u>Minor:</u>

- The authors make no mention of the padding used when smoothing. This can have a drastic impact on the calculated error, especially for large half windows. It could also have a different influence on spike distribution with high spike probability in the centre or at the periphery of the environment
- Line 163, the authors should also consider citing the following papers which are to my knowledge the first published report of rate maps (although in radial mazes).

  McNaughton, B.L., Barnes, C.A. & O'Keefe, J. The contributions of position, direction, and velocity to single unit activity in the hippocampus of freely-moving rats. *Exp Brain Res* 52, 41–49 (1983).

  Barnes, Carol A., Bruce L. McNaughton and John O’Keefe. “Loss of place specificity in hippocampal complex spike cells of senescent rat.” *Neurobiology of Aging* 4 (1983): 113-119.

- 461 “Generally, the error in place field detection was anti-correlated with map error (Fig 10; i.e., histogram) but this was not always the case (i.e., ASH).” This is not shown directly in the figure, the reader needs to integrate the results of figure 9 and 10 to deduce this. The author should show it in a figure
- 557: figure 12I does not exist, I think the author cite 12b instead
- In figure 12b the author does not comment on the fact that the estimated field radius with Ripley k is bimodal, notably for place field size under 250mm. This bimodality could influence the correlation reported in the figure.
- Figure 13: missing legend for e)
