# Supplementary material for: Estimating neuronal firing density: A quantitative analysis of firing rate map algorithms
Source: PLoS Comput Biol. 2023 Dec 27;19(12):e1011763. doi: 10.1371/journal.pcbi.1011763 (PMC10775984; doi:10.1371/journal.pcbi.1011763)
Supplement: Attachment — Submitted filename: Response to reviewers.docx [file pcbi.1011763.s014.docx]

# Response to reviewers

We thank the reviewers for their feedback and consideration. Detailed responses are included below in blue. Line and figure numbers correspond to the revised manuscript.

# Reviewer 1

Six different algorithms for binning up spatial (x,y) position and spiking data to create firing rate maps are evaluated and compared to a "ground truth" data set where the spiking pdf is known and to each other in terms of computation time, error (cf ground truth) and accuracy. The conclusion reached is that the bivariate histogram (which remains the most commonly used method for the last 50 years), plus some smoothing, is the optimal solution almost all of the time.

It's nice to see this kind of analysis collected in one place as the effects of various parameters on binning data are often overlooked in the literature, and if they are mentioned, they are usually buried across several papers. However, I have several concerns I would like to see addressed before recommending for publication.

## Major comments

1. One of the main difficulties in assessing the algorithms reported here is that as of this writing (01/08/23) the code was still not publicly available on Github (although I applaud the authors efforts to publish it openly eventually).

Apologies for this, the GitHub repository was not publicly visible. All code used for running simulations, analysis, and plotting is now available in the GitHub repository at <https://github.com/RoddyMGrieves/Rate-map-quantification>. The *rate_mapper* code used to generate firing rate maps using any of the methods described in this manuscript is available in the GitHub repository at <https://github.com/RoddyMGrieves/rate_mapper>.

2. Although the paper claims to be a quantitative assessment there is not a single statistical test conducted on any of the results. Graphing out the performance of the various methods is fine and gives the reader a qualitative sense of how well they do over a range of values but a quantitative comparison would be useful too.

Our main quantification is the relationship between error, computation time and mapping parameters such as bin size and smoothing strength, which can be found in Fig 9 and Table 1. We have also added statistical test results to the main text, in the summary/comparison results section [line 561] and post-hoc comparison results have been added to Fig 10a, b & d. Fig 11a contains a linear correlation which can be found in the figure legend and main text. Statistical quantification has been included in the new Supplementary figures S5, S7 & S8.

3. It is stated in the Introduction that the experimental sampling of position is often non-uniform. The position data generated here is fairly close to uniform, a desired end-state in many open-field recordings. However, some of the binning methods presented here are explicitly designed to deal with cases where sampling of one or more variables is non-uniform. It would therefore be useful to see how the different binning methods deal with positional data that is non-uniform i.e. where there are chronically *under-sampled* areas of the simulated environment.

We agree with the reviewer’s observation. To address this, we simulated an additional population of 64 place cells in 8-minute long sessions under three conditions: uniform sampling, goal-directed sampling (bias towards one spatial location) and thigmotaxis (bias towards walls). The main results did not vary greatly under these conditions except that error consistently increased across mapping methods in the goal-directed condition. We have added a section outlining the methods of this analysis (Methods: *Random walk*), a figure showing the results of this analysis as supplementary information (Fig S8). Additionally, we have added the following text to the main text:

[line 602]: *“Uneven trajectories, such as those caused by goal-directed navigation or thigmotaxis, did not change the results of the bin size and smoothing analyses (S8 Fig) although MISE did increase significantly when trajectories showed strong goal-directed biases.”*

## Minor comments

1. Abstract - line 24 - "The analysis of spatial neurons...", surely all neurons are spatial? Be more specific with your language - "neurons that exhibit receptive fields dependent on an organisms spatial location" or similar.

We have added the text suggested by the reviewer: [line 24] *“The analysis of neurons that exhibit receptive fields dependent on an organisms spatial location, such as grid, place or boundary cells…”*

2. Somewhere in the Introduction (around line 91 would be a good place) a list of the different mapping techniques should be provided

We have added the following line where the reviewer suggested: [line 92] *“The mapping methods we compare here are the bivariate histogram, averaged shifted histogram, adaptive smoothing and adaptive binning, and two kernel smoothed density estimate approaches.”*

3. You mention that Sturges rule is inappropriate for non-normal data, yet use it throughout the rest of the paper. Justify this.

In our introduction we discuss why Sturge’s rule and other rules-of-thumb are not appropriate [line 73]. Later in the manuscript we compute the rules-of-thumb values, but this is only for comparison with our findings, and as a possible explanation for the patterns observed in the MISE plots [line 427]. We do not use Sturge’s rule in our main analyses.

4. Simulated dataset - line 126 - are the generated trajectories actually unique? Looking closely at Figure 1E it looks like the shorter length trajectories are actually subsets of the longer ones.

The reviewer is correct; for speed and computational simplicity 64-minute duration sessions were generated and then clipped to shorter durations. This also ensures continuity between the durations (i.e., for each cell the 64-minute-long session represents the activity which would have been observed had the 16-minute long session been continued). For clarity the following lines have been added to the methods section:

[line 745] *“To investigate the effect of sampling we tested 4-, 16- and 64-minute long sessions, these were generated by simulating a 64-minute long session as described above and then clipping the sampling to the desired duration from the start of the session.”*

And to the results section:

[line 127] *“These walks simulated 64-minute-long sessions (Fig 1a & e) which were also clipped to 4- and 16-minute durations so that we could investigate the effects of recording duration and behavioral coverage.”*

5. Figure 1. It's unclear at this point what "variance in x and y" means (you have to dig into the text/ methods to find) and so it isn't really necessary - I think it defines how skewed the receptive fields are.

Increasing a field’s standard deviation in the x- **or** y-axis can produce fields elongated parallel to the x- or y-axis. However, variance in x **and** y can produce elongated fields with an orientation which is not parallel to the x- and y-axes. For clarity the following text has been added to the figure legend: [Fig 1] *“σxy gives the field’s variance in x and y and is used here to give fields an orientation oblique to the x- and y-axes.”*

6. Line 183 - you mention that the smoothing kernel used is Gaussian - there are other smoothing kernels, why use a Gaussian and not another? It needs justification in light of the authors statement in the Introduction that the shape of place fields are rarely normally distributed.

We employed a Gaussian kernel for the histogram and KSDE methods because they are the most frequently used shape of kernel in contemporary literature. The temporal KSDE approach employs a Blackman window and the averaged histogram employs a biweight kernel as these were the approaches described in the literature. The adaptive smoothing and binning approaches do not employ a smoothing kernel. In each case we justify the choice of kernel in the corresponding methods section [i.e. line 811, 847, 952 & 984].

Gaussian kernels are generally attractive because they are isotropic and their smooth shape does not introduce artefacts. In a MISE sense the shape of smoothing kernel actually has little effect on the resulting distribution (Zucchini, Applied smoothing techniques, Part 1 Kernel Density Estimation., 2003) – the bandwidth of the kernel has a larger effect, which is why we concentrated on smoothing strength/bandwidth in our analysis.

7. This is a personal preference for the order in which the Figures are presented but you present schematics of the histogram method then one concerning a qualitative assessment of the different methods, then figures, in order, of the adaptive smoothing and then KSDE methods. Surely it's more logical to present each of the methods in order and *then* the figure showing the qualitative assessment of the different methods?

We agree with the reviewer and this figure has been moved to the beginning of the quantitative analysis section [now Fig 6].

8. Line 201-2 - makes it sound like smoothing has occurred before *and* after. Tighten the language up.

This language has been clarified: [line 196] *“In the vast majority of cases spike and dwell time maps are smoothed and then used to produce a firing rate map which does not require its own smoothing. Alternatively, unsmoothed spike and dwell time maps can be used to produce a firing rate map which then has smoothing applied to it.”*

9. Line 224 - "One issue that has been raised..." reference this immediately after.

This has been added, thank you [line 220].

10. Line 241 - any speculation as to why this has never been implemented?

The averaged shifted histogram (ASH) has not really been implemented widely in any field. Research progressed from histograms to kernel-based methods and the ASH was never fully explored. We included it here because it bridges the gap between these approaches and offers some unique advantages, such as obtaining very high-resolution maps with little computational cost. Having a more detailed map may be useful for researchers who wish to estimate field size or shape more accurately.

11. You use the same spike/ position data for Figures 2 & 5 but not for the other examples of how the binning methods work. It would be nice to have the same spiking / position data for all of them to facilitate an comparison with the eye of truth.

We agree with the reviewer. The methods figures (Fig 2-5) now all use the same example spike and position data.

12. The introduction to adaptive smoothing (line 306+) diverts into talking about 3-dimensional binning which is off-topic. You should limit the discussion to papers binning up 2D data.

The only electrophysiology papers to use an adaptive binning approach are those investigating freely flying bats, thus the only implementations described in the literature are three-dimensional. However, adaptive binning is K-nearest neighbor density estimation by another name, which has been described in two-dimensions previously (Silverman, 2018; <https://doi.org/10.1201/9781315140919>). For context we would like to briefly discuss the implementations in the literature, although we have now reduced the detail of this in text [line 296].

Despite this, I found the explanation unclear as to what the result of the binning process is. It sounds as though you end up with bins of unequal size, which would make comparison between maps with different positional sampling impossible. An explanation of how data is allocated to a bin should be more explicit i.e. what you end up with is a regularly spaced, but unevenly sampled, grid.

The reviewer is correct, the adaptive smoothing and binning methods both expand or contract local bins to accommodate the local density of the data. In this way, maps contain bins that are regularly spaced but based on differently sized regions of underlying data. The aim of this approach is to increase the accuracy with which local firing rates are estimated by compensating for under sampled regions. This should increase the validity of comparisons between maps with different positional sampling, not reduce it. We have added text to both methods for clarity: [line 862 & 895] *“After iterating across all bins, the result is a firing rate map with a regularly spaced grid of values, based on differently sized regions of underlying data which may also be overlapping.”*

13. Line 328 "...though..." should read "through".

This has been corrected, thank you [line 316].

14. Line 340 "...the most widely adopted approach..." by whom? Provide references.

We have added the appropriate references here [line 329].

15. Line 357 "...firing rate map" should more accurately read "discretized map"

This has been corrected, thank you [line 346].

16. Fig 8. Returning to a point made earlier, there is no comparison being conducted here beyond a visual one. This figure is a good example - correlations could be performed between the 3 different firing rate map types produced and an ANOVA could be conducted on the computation time.

We have added statistical test results to the main text [line 560] and post-hoc comparison results have been added to Fig 10a, b & d.

17. Lines 433-45 I'm not sure how the bin sizes were calculated here; my calculation of Sturges rule gives bin sizes of 17 and 19mm for 16 and 64 minute recordings respectively which intuitively feels right as opposed to the 70 and 63mm sizes given in the text (these are pretty large bins given the usual 2-3cm employed in most experimental papers). Similarly, my calculation of the Friedman-Diaconis rule didn't agree (I got 33 and 20mm). I also feel as though there was no rationale provided as to why one rule of thumb was used over another. Please provide one.

Sturge’s rule estimates the required number of bins, but this has to be used in conjunction with the bounded support of the data (1200 mm) to derive a bin size in mm: i.e. 1200 divided by the 17 or 19 values calculated by the reviewer gives the 70 and 63 mm values found in the manuscript. We do not provide a rationale for either rule of thumb because they are only reported for comparison to our results, we do not recommend using them for spike & position data.

18. State why an "abundance of empty bins is undesirable".

An abundance of empty bins is often undesirable when investigating a neuron’s spatial activity because the purpose of a firing rate map is to estimate a cell’s activity at each spatial location but empty bins represent locations for which we do not have an estimate of the cell’s activity.

19. Figure 10 I'm slightly unclear what some of the numbers mean. What does an error of 0.5 mean? 0.5%? 50%? Or something else? The same applies to the "proportion of map empty" plots.

We have added text to the results section which points to the methods section describing this measure: [line 1014] “The place field detection error rate was calculated as the difference between the number of place fields detected in the firing rate map and the number detected in the actual spike probability map”.

20. Do the values in Table 1 need to be accurate to 3 decimal places? Seems like overkill

Some of the values are small enough that they only have a value at the 3^rd^ decimal place. Small inaccuracies in these values can also have a large impact on the regression fit. For these reasons, and for consistency, we include all of the values to 3 decimal places.

21. Figure 12 - it wasn't clear where/ how the centre of the circles were placed.

For demonstration, the circles (in panel a left) are centered on a location close to the center of the place field, this location provides a visually clear demonstration of the method in the following panels. However, as stated in the figure legend: [Fig S6] *“Ripley’s K function is related to this example, but it is repeated across many different locations in the environment and the results are averaged.”* Thus, in practice this procedure is repeated across the environment and the center of the place field does not need to be known.

22. I'm unclear on the preoccupation with the proportion of empty bins (especially given that the positional sampling is almost perfectly uniform) cf Figure 13d

Empty bins are something we want to minimize because they are locations at which we have no estimate of a cell’s activity. However, this must be balanced with map accuracy. For instance, a firing rate map made of a single bin spanning the entire environment will have no empty bins, but is also not useful for spatial analyses. Thus, we want to minimize error while also minimizing the number of empty bins. Additionally, minimizing empty bins is a priority of the adaptive mapping methods, so we sought to include it in our comparisons.

23. Figure 13e - needs colour bar

This has been added, thank you [Fig. 10].

24. Line 628 - "a weak, but significant, decrease..." no statistics are reported to back this statement up

The correlation coefficient and p-value are reported in the figure legend [Fig. 11a] but we have also added this to the main text [line 635].

25. I don't think it's necessarily that "the field has recognised the increased accuracy afforded by smoothing" that has led to the adoption of Gaussian smoothing kernels. If you have an a priori reason that the underlying response function is Gaussian (and we do with place fields for eg) then the raw data should be smoothed with a kernel that reflects that assumption, thus emphasising the underlying response and de-emphasising the things you don't want.

We agree with the reviewer that using smoothing emphasises the cell’s underlying response while de-emphasising unimportant features, this increases map accuracy which is one of the reasons kernel-based methods are generally found to be more accurate. We think this is one of the driving reasons the field has adopted Gaussian smoothing as the norm.

26. Line 661 "balance" should read "balanced"

This has been corrected, thank you [line 666].

# Reviewer 2

In the current manuscript, Grieves provides a thorough analysis of several methods for quantifying position-selective firing of recorded neural data. I find the work to be detailed and well-described. Despite the fact that this is a rather mature field, I am unaware of a similar study which directly compares place field quantification methods and criteria, making this a novel and important study.

## Major comments

1. I have only one concern, which regards the manner in which spiking was generated from the ground truth place fields. The author uses a Poisson distribution to generate spike patterns. However, Fenton & Muller (PNAS, 1998) showed that firing of place cells is rarely well described by a Poisson distribution. Rather, individual passes through place fields display “overdispersion” (at least, in some cases and/or environments). Overdispersion makes quantifying place fields particularly challenging for cells with relatively low max firing rates or for experiments with limited behavioral coverage of the environment.

Thus, I would like the author to repeat some of the core analyses using spikes generated from an overdispersion model (see Olypher et al, Neuroscience, 2002). I don’t think it’s necessary to repeat all analyses, only those where the variability introduced by overdispersion would be most impactful: low peak firing rate place fields and short-duration recordings.

We agree with the reviewer’s observation. To address this, we simulated an additional population of 64 place cells in 4-minute long sessions under two conditions: no overdispersion and heavy overdispersion. Overdispersion was implemented following an approach inspired by Olypher et al. (2002) – the cell’s spiking was modulated by a time varying signal that introduced 80% spike variability at 1.5 Hz intervals. The main results did not vary greatly under these conditions (Fig S7) except that the optimal smoothing parameter consistently increased across mapping methods. We have added a section outlining the methods of this analysis (Methods: *Spiking*), and a figure showing the results of this analysis (Fig S7). Additionally, we have added the following text to the main manuscript:

[line 597] *“These results did not vary greatly when cells were simulated to include heavy overdispersion (Fenton and Muller 1998; Olypher et al. 2002), although the balanced and minimum error solutions consistently shifted towards significantly stronger smoothing (S7 Fig). This increased smoothing requirement likely reflects the larger uncertainty associated with overdispersion and should be taken into account when analyzing recording sessions of a short duration and when overdispersion is anticipated.”*

2. An additional minor critique is that the author uses the term "we" throughout the manuscript even though it is a one-author paper. I believe this should be changed to "I" or rephrased to remove the subject pronoun entirely from these sentences.

I much prefer to use 'we', as in "the author and the reader", than ‘I’ which is rarely used in scholarly writing (but is admittedly becoming more common). Removing subject pronouns completely also leads to a lot of awkward sentence structures. Since this is a stylistic choice and PLoS does not have an author guideline about personal pronouns I would prefer to continue using ‘we’.

# Reviewer 3

This manuscript reviews existing and new methods and parameters used to generate firing rate maps of spatially modulated neural activity. In particular, the author thoroughly explores the impact of those methods and choice of parameters on how the rate maps correspond to the underlying spike distribution, which is derived from simulated data.

Overall, the analysis is rigorously performed, and the results convincingly demonstrated. The comparisons performed in the paper are informative for the spatial cognition field. However, I have concerns about the novelty and the impact of the results of this paper. In terms of novelty, the author showed that the bivariate histogram with spatial smoothing was the preferred choice in the majority of cases, but this is already the most commonly used method to compute rate maps. Similarly, in terms of impact, the author shows a method to optimise the parameters used when computing rate maps, but those optimal parameters are in the range most commonly used in the literature. Furthermore, the fact that this study is only performed on synthetic data makes it difficult to gauge the efficiency of this method on real datasets.

## Main points

1. The way the author quantifies the rate map error is difficult to reconcile with a tangible measure. It would be helpful to provide other measures of similarity (correlation, mutual info, population vector distance, …) between the ‘true’ rate map and the computed rate maps. In its present form, the MISE is adequate to investigate the point that the author is making (eg: best method and parameters for rate map computation) but it is difficult to relate the MISE and thus the variation of method and parameters to concrete and more classic similarity measures. The author uses the place field detection, compute time, and proportion of empty bins, but adding other measures, classically used in the literature would strengthen the message and scope of the method detailed in this paper. In its present form, I am circumspect about the impact of these optimisations on a real dataset.

Mean integrated squared error (MISE) has been the primary measure used to quantify and optimize density estimation techniques since the 1950s, so we have chosen to keep this as the primary measure of similarity throughout the manuscript. However, we have added a comparison between MISE, Pearson correlation and Euclidean distance in figure S5. These similarity measures are all extremely consistent with each other. Mutual information is difficult to apply to our wide range of map sizes, with wildly different firing rate scales due to bin size, and is also quite slow to calculate which has prevented us from including it. We also do not store the firing rate maps created during our analysis due to the disk space this would require, so a population vector distance is not possible in our analysis.

2. This analysis is performed on synthetic data, where the author fitted as much as possible various parameters of real datasets (see methods). However I am concerned about the robustness of these results on real datasets. Notably, with different place field size (more variation than presented in this paper), different geometry of the environment (linear/star mazes, multiple rooms, very large environments....). The demonstration of the best method to bin spike data needs a synthetic dataset in order to access the underlying spike probability. However, to optimise for the detection of place fields, compute time, and empty bins (fig 10-13), in some cases, the author does not necessarily need the spike probability distribution. In these cases, it would be interesting and easy for the reader to grasp the effect of the parameter optimization on a real word dataset. In a real dataset, the place field size, number, firing rate and distribution will vary much more than for the simulated place cell population of this paper ( random distribution of place fields , same firing rate, different place field size in a single cell,... ). All these discrepancies, notably the difference in firing rate and size of place fields have the potential to strongly influence the optimal parameter choice (optimal smoothing,... ).

Using a real dataset we can only optimize for computation time and empty bins. Without knowing the underlying spike probability distribution we cannot calculate MISE, or quantify place field detection error because we cannot know the ground truth of how many fields a cell expresses. For these reasons we adopted a simulated dataset which attempted to replicate as much of the variation observed in real place fields as possible – our simulated cells do have different firing rates, numbers of fields and fields of varying size and shape within a cell. These distributions were chosen to reflect those observed in real datasets. We have also included supplementary analyses which include overdispersion (Fig S7) and under sampling (Fig S8).

However, to further test the robustness of our results on real data we have used the equations presented in Table 1 to calculate parameters for firing rate maps of real place cells (retrieved from the dataset of Grieves et al. 2020; <https://doi.org/10.1038/s41467-020-14611-7>) which are shown in Fig S9.

3. 46 “These parameters can have a huge impact on the map created”, The author shows that this is the case in theory. However, the author shows in Fig 14 that for previous work the solution is very close to the balanced solution. I thus wonder how big is the difference in place field detection, MISE,... in the range of parameters classically used in the field. According to figure 13, the difference might be small.

Fig 11b shows the parameters used in the literature superimposed on the MISE map for each approach. In this figure we can see that while some (*) points fall towards higher error areas, researchers generally choose parameters which are low-error. These values can also be compared to the place field detection error, computation time and empty bin plots in Fig 8, which are plotted on the same axes. Doing this, we can see that researchers have generally chosen parameters which fall in the low field detection error, fast computation time, low empty bin regions – which makes sense because their parameters are close to the balanced solution which attempts to minimize all of these.

## Minor points

1. The authors make no mention of the padding used when smoothing. This can have a drastic impact on the calculated error, especially for large half windows. It could also have a different influence on spike distribution with high spike probability in the centre or at the periphery of the environment

For the histogram, ASH and adaptive methods maps were padded with zeros to accommodate the kernel. For the KSDE approach we used the “reflection” boundary correction method. We have added text to the corresponding method sections to clarify this [i.e. line 805, 841, 942].

2. Line 163, the authors should also consider citing the following papers which are to my knowledge the first published report of rate maps (although in radial mazes).

McNaughton, B.L., Barnes, C.A. & O'Keefe, J. The contributions of position, direction, and velocity to single unit activity in the hippocampus of freely-moving rats. Exp Brain Res 52, 41–49 (1983).

Barnes, Carol A., Bruce L. McNaughton and John O’Keefe. “Loss of place specificity in hippocampal complex spike cells of senescent rat.” Neurobiology of Aging 4 (1983): 113-119.

We have added these citations at the beginning of the histogram results section where there is a brief background to the approach [line 168].

3. 461 “Generally, the error in place field detection was anti-correlated with map error (Fig 10; i.e., histogram) but this was not always the case (i.e., ASH).” This is not shown directly in the figure, the reader needs to integrate the results of figure 9 and 10 to deduce this. The author should show it in a figure

This has been corrected and the following text added: [line 452] *“Generally, the error in place field detection was correlated with map error (Fig 8; histogram: r = 0.25, ASH: r = 0.78, Adaptive smoothing: r = .90, tKSDE: r = 0.54, KSDE: r = 0.72, Adaptive binning: r = 0.98).”*

4. 557: figure 12I does not exist, I think the author cite 12b instead

This has been corrected, thank you.

5. In figure 12b the author does not comment on the fact that the estimated field radius with Ripley k is bimodal, notably for place field size under 250mm. This bimodality could influence the correlation reported in the figure.

The Ripley k analysis is by no means a perfect answer to this issue, we present it here as one possible avenue for field size estimation, although it will require further research. For this reason we have moved these results to Supplementary Information (Fig S6). We have also included a short discussion on these results: [line 548] *“Using this method on our simulated place cell dataset, we found a satisfactory correlation between the actual and estimated place field sizes although this could be improved (S6b Fig; r = 0.50, p = 1.3 × 10-50, Spearman’s correlation between estimated and real field size). Additionally, the field sizes estimated using this approach are often bimodal (S6b Fig; modes somewhat separated by the solid black line), the reason for this is unclear but may be due to edge effects occurring when fields are cut off by a boundary. Further research could look to improve on these kinds of density metrics.”*

6. Figure 13: missing legend for e)

This has been corrected, thank you [Fig. 10].
