# Supplementary material for: Estimating neuronal firing density: A quantitative analysis of firing rate map algorithms
Source: PLoS Comput Biol. 2023 Dec 27;19(12):e1011763. doi: 10.1371/journal.pcbi.1011763 (PMC10775984; doi:10.1371/journal.pcbi.1011763)
Supplement: Attachment — Submitted filename: response to reviewers.docx [file pcbi.1011763.s015.docx]

# Reviewer #1

I thank the authors for making the amendments to the MS and making the data and code-base available.

We thank the reviewer for their comments and insight.

# Reviewer #2

The authors have fully addressed my original concerns.

We thank the reviewer for their comments and insight.

# Reviewer #3

## Summary

This manuscript represents an updated version of a prior submission, offering a comprehensive review of both traditional and novel techniques for creating firing rate maps of spatially modulated neurons. The author has extensively investigated how these methods and their parameter choices align the computed firing rate maps with the underlying, simulated, spike distribution data. Building on the original manuscript, the author has conducted detailed analyses and compiled a valuable summary of various approaches for producing firing rate maps of spatially modulated neurons. This revised version also addresses several minor issues raised earlier and includes additional insightful analysis. Despite these enhancements, I still have reservations regarding the paper's originality, practical value, and overall impact.

We thank the reviewer for their thoughtful comments. We believe that our study is original, as it is to our knowledge the first to quantify the accuracy of rate mapping methods (please see also reviewer 2’s previous comments: “Despite the fact that this is a rather mature field, I am unaware of a similar study which directly compares place field quantification methods and criteria, making this a novel and important study.”). We also believe our shared code has a high practical value as neuroscientists can use it to assess the optimal mapping parameters for their own experimental conditions. In terms of impact, our study offers the potential to validate in a quantifiable manner a large range of methods used in the spatial cognition literature so far (1983-2023) and the numerous upcoming studies of the field would strongly benefit from such validation of their mapping methods.

One of the main results of this paper is that the bivariate histogram method, combined with spatial smoothing, has been identified as the preferred method in the majority of scenarios. However this method is already widely acknowledged as the simplest and most commonly used approach for constructing rate maps.

Indeed, we see our quantitative validation of the most widely used rate mapping approach as a positive outcome of our study, reinforcing trust in past results upon which many of the current and future experiments in the field are built. Please also note that the variability in the use of the bivariate histogram is still quite large in the literature (see new Fig S10) and a large portion of the studies do not employ smoothing (top part Fig 11B), which we found improves map accuracy [line 410].

The parameter optimization could also lack a “real word” use case in my opinion. The scale of the differences reported in the paper (computation time, MISE error,...) is really small, and the condition of this demonstration is very (too?) controlled and is not shown to hold with the variety of arena shape and scale but also the variety of place field size and other factors as mixed selectivity for example.

The scale of the MISE/correlation differences is not small (see answer to main comment 1 below). Computation time varies by orders of magnitude between mapping methods and, as we outline in the manuscript, in cases where maps are generated *en masse*, such as shuffle procedures, even a small difference can amount to a massive time loss. Finally, while we tested the robustness of our results for 3 different recording durations and 3 different field sizes, the reviewer is correct that we did not test different arena shapes and we chose to focus on pure spatial coding instead of mixed selectivity for the sake of simplicity; however, our methods can easily be extended to test the influence of other parameters by interested researchers.

Overall, the principal merit of this paper lies in its methodical compilation and examination of various methods for calculating rate maps, alongside a review of relevant literature in the field of spatial cognition. This comprehensive approach makes the paper a valuable resource for researchers in the field. However, I maintain reservations regarding the strength of its central message. The differences it reports are quite minimal, even using synthetic data. Consequently, I remain sceptical about the practical application and effectiveness of the parameter optimization strategies proposed in this paper in real-world scenarios.

We thank the reviewer again for their comments and insight. We have now quantified the reported differences and we hope the reviewer will now be convinced that they are significant. However, we would like to point out that even if the differences between map accuracy for different parameters were not substantially different, such a result would also have been of interest to the field, providing the first quantification that even a range of varied parameters still gives accurate results. In addition, we have designed our simulations to mimic as closely as possible real-world electrophysiological data, including realistic trajectories, overdispersion, uneven sampling, different recording durations, field sizes, field shapes, field number and place cell firing rates.

## Main comments

1. Answer to main point 1: Despite the MISE being “the primary measure used to quantify and optimize density estimation techniques since the 1950s” I think it still fails to help the reader to grasp the extent of the difference between the different parameters/methods studied here. The MISE being in the range of 10-12 at worst (see fig S5) means that the average difference between the observed and synthetic rate map is ~10-6 Hz. This difference is very small and unlikely to make a significant difference in real, less controlled dataset. This lack of consequential effect of the parameter optimization is reflected by the difference (best vs worst case) of correlation: +- 0.1 , euclidian +- 0.07 Hz .

Please note that the MISE and Euclidean values reported in text are computed on two probability density functions, hence their small values, they are not in units of Hz. To clarify this, we have added the following to the main text [line 398] “note that MISE is in units of integrated probability squared, not firing rate”.

Additionally, the colorbars in Fig S5 do not reflect the full range of data values. Figure 7, plotted with method-specific color axes is shown below, and gives the false impression that the KSDE and tKSDE methods are comparably accurate, when tKSDE actually exhibits much higher errors. For this reason, we used a consistent color axis across plots which allows comparisons between methods. We have added text to the legend of Figs 7, 8, S1, S4, S5, S7 and S8 to explain this, i.e. [line 453] “Note that plots are shown using a consistent color axis which may not span the full range of data values for every method”. In Fig S5 this means that average correlation values for the histogram method in reality range between 0.25 and 0.96 and not just ±0.1.

This method and parameter changes make a difference on synthetic data (place field detection,....) but are unlikely to make a significant difference on a real dataset with more noise (SNR, non gaussian field, inhomogeneous sampling, environment shape,...).

As described above, we have attempted to mimic, as close as possible, the parameters and variability of real-world data. 1) To mimic signal to noise ratio our simulations include out-of-field firing which is modelled as random noise added to a cell’s activity. Future tests will need to be made to investigate the effects of different background firing rates seen in other spatial cell types. Spikes are also randomly shifted forwards and backwards in time to mimic the effects of phenomena such as phase precession and theta sequences. 2) The reviewer is correct that our fields are modelled as multivariate Gaussians, however, they can and do overlap to form non-Gaussian fields. 3) We have previously tested the effects of inhomogeneous sampling (Fig S8), these results are described in the main text, section “Overdispersion and uneven sampling”. 4) Environment shape is a factor which will need to be investigated in the future, it is unlikely any of the properties we have shown here would change in a differently shaped open field arena, but for complex alleyway mazes, non-isotropic smoothing kernels that more closely fit to the underlying position sampling will likely need to be developed. To highlight these potential limits of our method, we have added a paragraph to the discussion in the “Limitations and further research” section [line 706].

2. For Mutual Information, a quick test seems to show the calculation of MI should be barely longer than calculating the correlation between two vectors (with a size comparable to the ones studied here).

We have now added Mutual Information to Figure S5, based on a smaller subset of 64 place cells. It is broadly consistent with the other error metrics. Please also note that the implementation we have adopted for computing mutual information (<https://www.mathworks.com/matlabcentral/fileexchange/13289-fast-mutual-information-of-two-images-or-signals>), the fastest that we found, is 55× slower than the correlation implementation (Matlab’s inbuilt *corr* function): 5.5ms vs 0.1ms, averaged over 100 iterations on 100×100 bin maps.

3. Answer to main point 1: Concerning the population vector distance or any population vector analysis suggested previously, storing rate maps should be a problem with modern computers. Storing ~1k rate maps with the maximum binning size of this study should not even take a gigabyte of RAM (in float64).

On the contrary, 1k ratemaps each with 1200^2^ bins (1440000; the smallest bin size used) would require: 1e3*(1.2e3^2)*64*1.25e-10 = 11.52Gb of space. All ratemaps for the histogram method alone would require: 256 cells × 17 smoothing values × 18 bin size values (N bins in each map) × 3 field sizes × 3 session duration parameter values or 256*17*sum(ceil(1200./[1 2.5 5 10:10:100 160 240 320 640]').^2)*3*3*64*1.25e-10 = 548.5Gb of space.

For simplicity we decided to make our pre-planned comparisons and discard these maps rather than store them. Computing a population vector analysis would require recomputing and storing the entire ratemap dataset, which would take weeks of computation. As we are unsure that computing this additional quantification of rate map accuracy (on top of MISE, Pearson r, Euclidean distance and mutual information) would add significantly to the manuscript, we chose not to implement it. In addition, the current manuscript is concerned with how to construct an accurate ratemap on a single neuron basis, not at the population level. It would be interesting to know if the best ratemaps for replay analyses or position decoding are the same as for spatial analyses but this is outside the scope of the current manuscript.

4. Answer to main point 2: This answer illustrates some of the previous criticisms I had on the lack of robust methods to grasp the real advantage of the methods and parameters on real data. Effectively, real data will not allow access to the underlying spike probability distribution and thus make the comparison with MISE impossible. However, the other comparison possibilities suggested by the author are not satisfactory as they heavily depend on the machine used for the analysis and the code implementation (language, CPU/GPU, various optimisations,...). To complement the additional illustration shown in figure S9, the author could quantify the error of place field detection, the MISE or an other distance metric between the worst parameter choice reported in the literature and the minimum or balanced parameter.

We thank the reviewer for this suggestion. In a new supplementary figure S10, we select different parameter combinations from the literature values, including the one with the highest average error, quantify and compare the MISE associated with each and show an example of what a firing rate map generated with these values would look like. We conclude that studies show a wide range of variability despite clustering around the balanced solution, and that place field detection was accurate across the parameter combinations reported in the literature [line 646].

5. Answer to main point 3: Effectively, it would be interesting to correlate the MISE error (Fig 11b) and the variable compared in Fig 8. I encourage the author to show such figures. With an indirect comparison, the colormap does not allow an accurate comparison.

The correlation between MISE (Fig 11b) and place field detection error (the main variable shown in Fig 8) is already provided in text: [line 462] “Generally, error in place field detection was correlated with map error (Fig 8; histogram: r = 0.25, ASH: r = 0.78, Adaptive smoothing: r = .90, tKSDE: r = 0.54, KSDE: r = 0.72, Adaptive binning: r = 0.98).”

Furthermore, as the author highlights, only a handful of studies chose parameter falling in the high error area and if so these points seems to fall in low place field detection error (max ~15%, with the bivariate histogram, it might be less but the colormap does not allow to distinguish).

It is unclear to us what the reviewer suggests here, but to quantify the differences in place field detection across the range of literature parameters, in a new supplementary figure S11, we select different parameter combinations from the literature values and quantify the place field detection error, computation time and the proportion of missing values associated with each. In addition, we have added the following to the main text: [line 650] “Place field detection was also very accurate across the parameter combinations reported in the literature (S11 Fig), this may be because place field detection represents only a very rough measure of map accuracy, or because researchers using firing rate maps qualitatively optimize them for accurate place field detection.”

## Minor comments

1. Answer to minor point 1: The use of zero padding “to reduce edge effects” is incorrect. Zero padding rate maps with non-zero out of field firing (most if not all spatial rate maps) will introduce a sharp transition at the location of the edge. This might not be a problem on synthetic data, but in my experience the mirroring or reflect option is preferable. No option will be a perfect/correct option as the data is not recorded outside of the arena but the phrasing is incorrect. I highly encourage the author to use another padding method and if not to rephrase the modification (line 850, 909, 952) saying that padding removes edge effect.

Without any padding edge effects would be more pronounced, which is what the sentence is referring to. However, the reviewer is correct that for cells with non-zero out-of-field firing this would create a sharp transition at the edge of the map. To highlight this in the manuscript, we have added the following text to the methods section: [line 838, 882 & 924] “We chose zero-padding because it most closely represents the actual data values collected without attempting to extrapolate spatial activity outside the sampling range. However, other padding methods such as reflective or symmetric padding may be preferred, especially when the out-of-field firing rate of a cell deviates significantly from zero, and these can be implemented using the code provided alongside this paper.”

2. Answer to minor point 5: The term "satisfactory" does not describe objectively a correlation between two variables.

We have substituted “satisfactory” for “high positive” [line 560] following the recommendations in Table 1 here: Mukaka (2012; <https://www.ncbi.nlm.nih.gov/pmc/articles/PMC3576830/>) *A guide to appropriate use of Correlation coefficient in medical research*.

Furthermore, "mode somewhat separated": the author could test his hypothesis - that the bimodality is caused by cut field - easily by selecting cells with place fields at the centre of the maze.

We thank the reviewer for this comment, which allowed us to improve our method for computing Ripley’s K. We previously followed the derivative improvement proposed by Kiskowski et al. (2009; <http://dx.doi.org/10.1016/j.bpj.2009.05.039>), however, upon inspection we have realized that this does not appropriately extend to place cells because H’(r) rarely crosses below the -1 threshold proposed by Kiskowski et al. (2009). Field size is overestimated when this fails to occur, which led to the bimodality and inaccurate overall estimation observed previously. We removed this analysis step, and the results no longer show the bimodal effect. The correlation between the actual and estimated field size has also improved from 0.50 to 0.71 (see Fig S6b). A density plot has been included in Fig S6b. The methods section for this analysis has also been updated (Methods: *estimating field size*).

3. The figure linked to the last version of the file are extremely blurry

The figures appear to have been compressed by PLoS’s submission system. The figure files provided to the submission system were high resolution (300dpi) and verified beforehand by the PACE preparation system. I have added a note about this to the Editorial team in my cover letter.

4. The text in some figure is very small and could easily be increased to improve readability (eg: in Fig 2: text in the top left of the environments)

This text, and corresponding text in figures 2-5, has been increased in size.
